# Supplementary material for: Technical Development and In Silico Implementation of SyntheticMR in Head and Neck Adaptive Radiation Therapy: A Prospective R-IDEAL Stage 0/1 Technology Development Report
Source: medRxiv. 2024 Sep 30:2024.08.29.24312591. Originally published 2024 Aug 31. Preprint. [Version 2] doi: 10.1101/2024.08.29.24312591 (PMC11383512; doi:10.1101/2024.08.29.24312591)
Supplement: Supplement 1 [file media-1.pdf]

Supplementary Materials

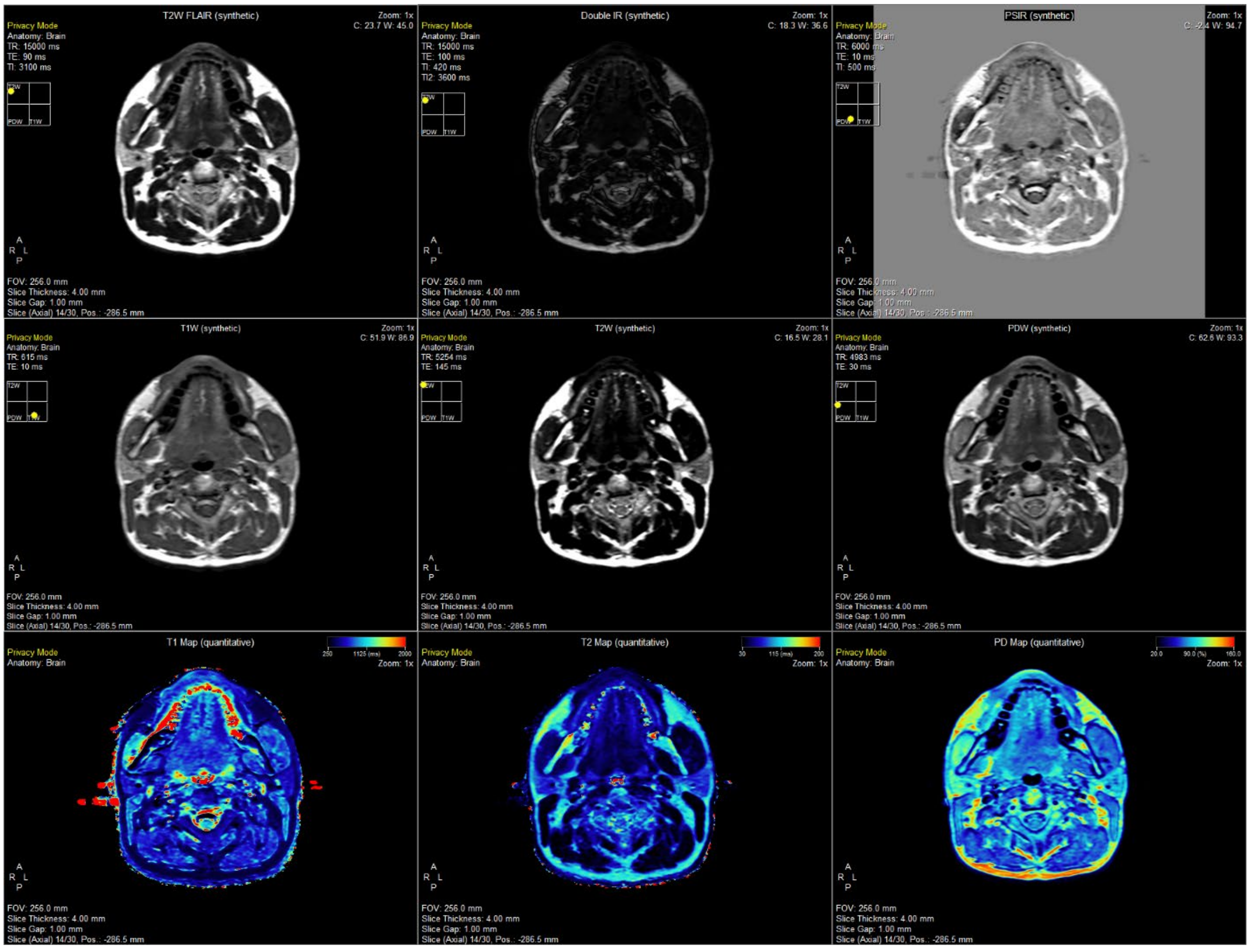

Figure 3-S1. Demonstration of the SyMRI post-processing package offered by SyntheticMR in Volunteer 1 on the MR-Sim scanner.

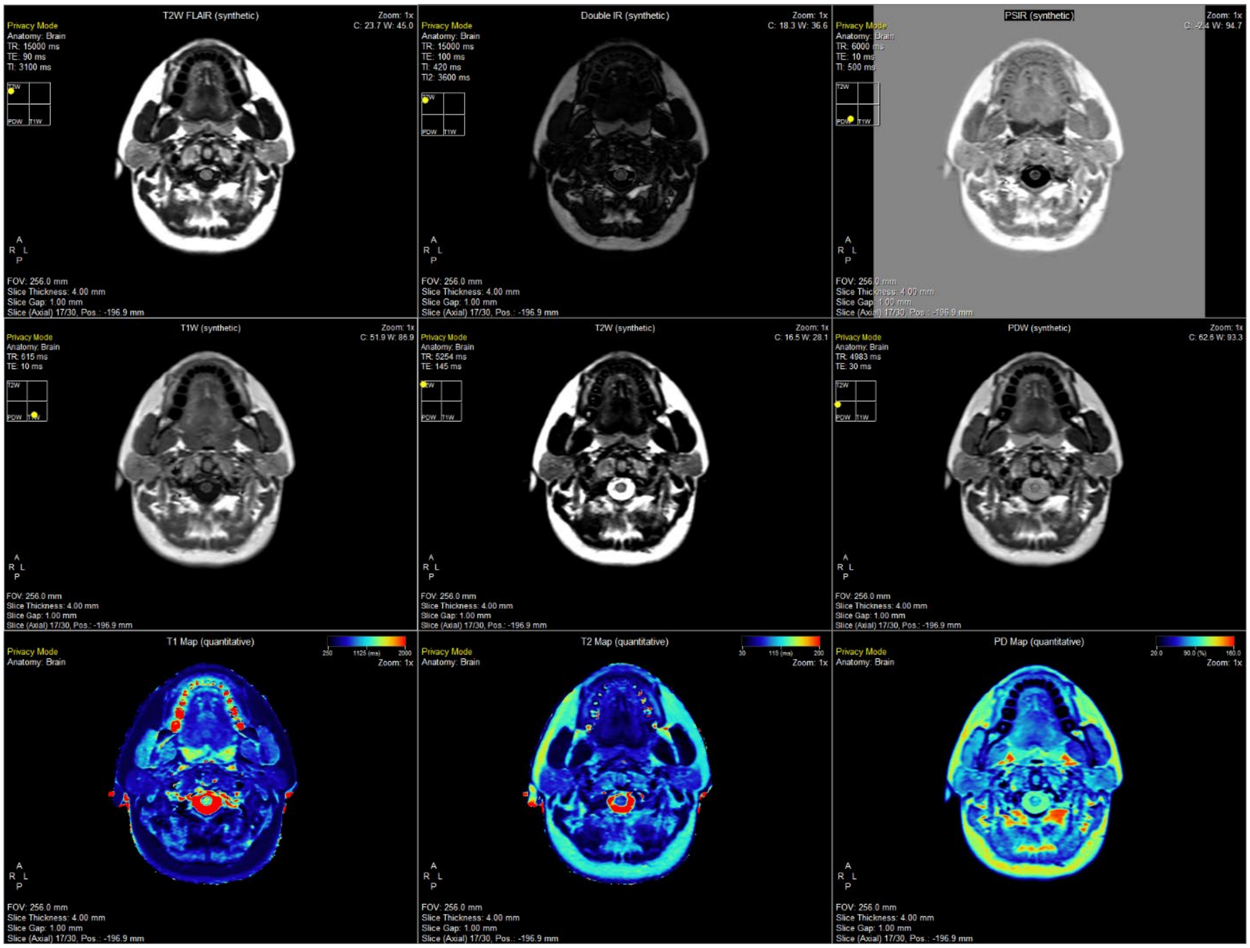

Figure 3-S2. Demonstration of the SyMRI post-processing package offered by SyntheticMR in Volunteer 2 on the MR-Sim scanner.

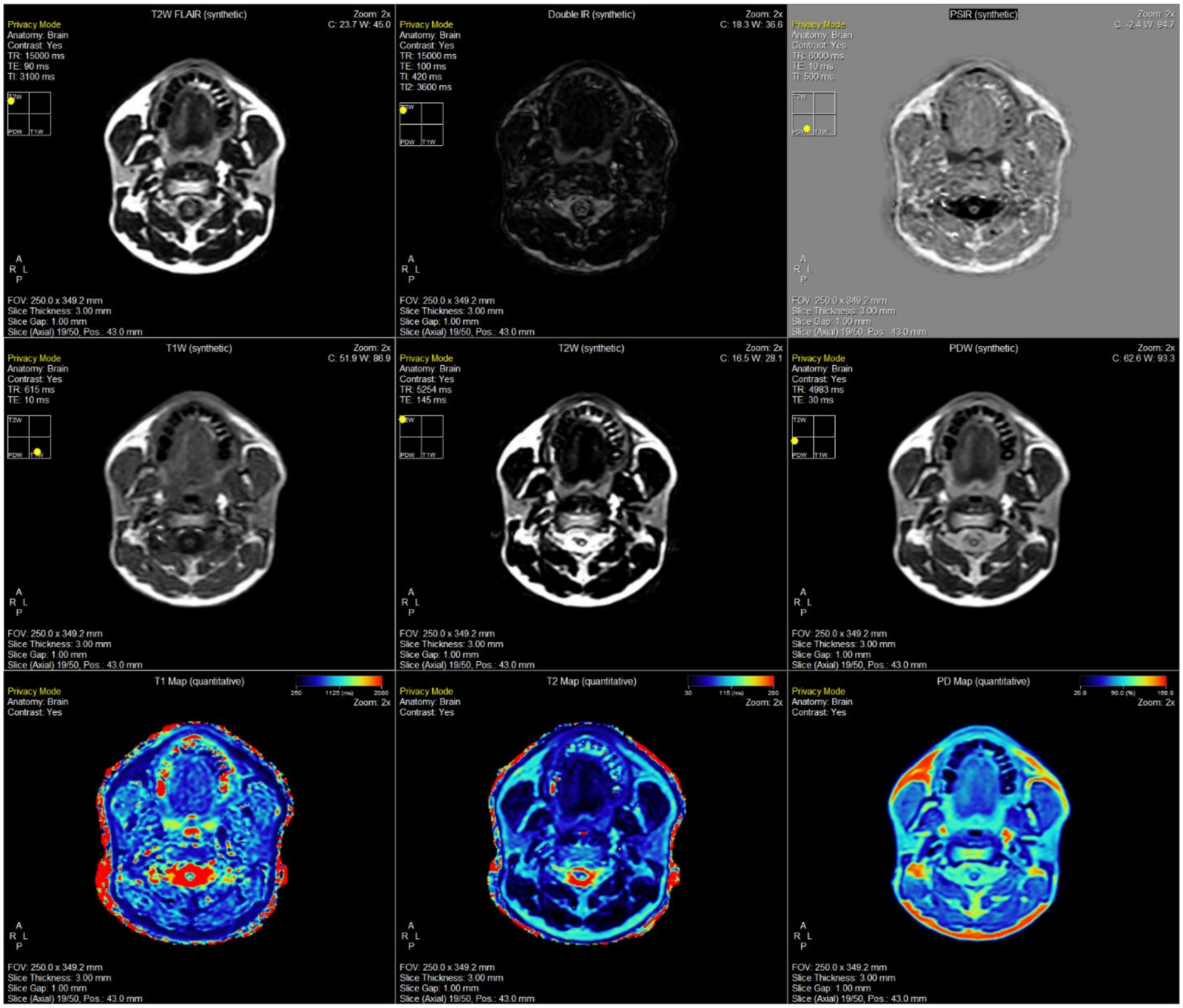

Figure 3-S3. Demonstration of the SyMRI post-processing package offered by SyntheticMR in Volunteer 1 using the coarse sequence on the MR-Linac scanner.

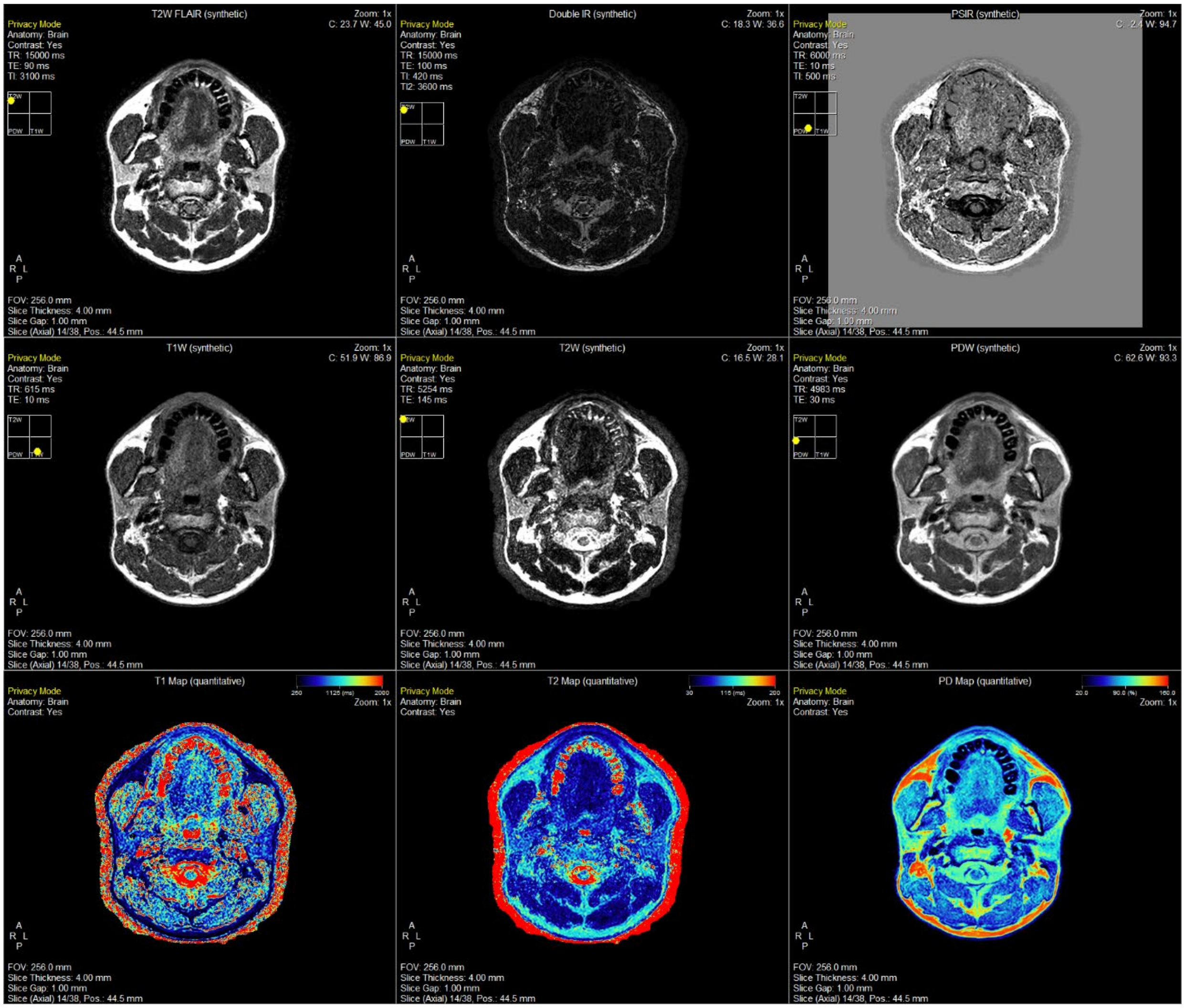

Figure 3-S4. Demonstration of the SyMRI post-processing package offered by SyntheticMR in Volunteer 1 using the fine sequence on the MR-Linac scanner.

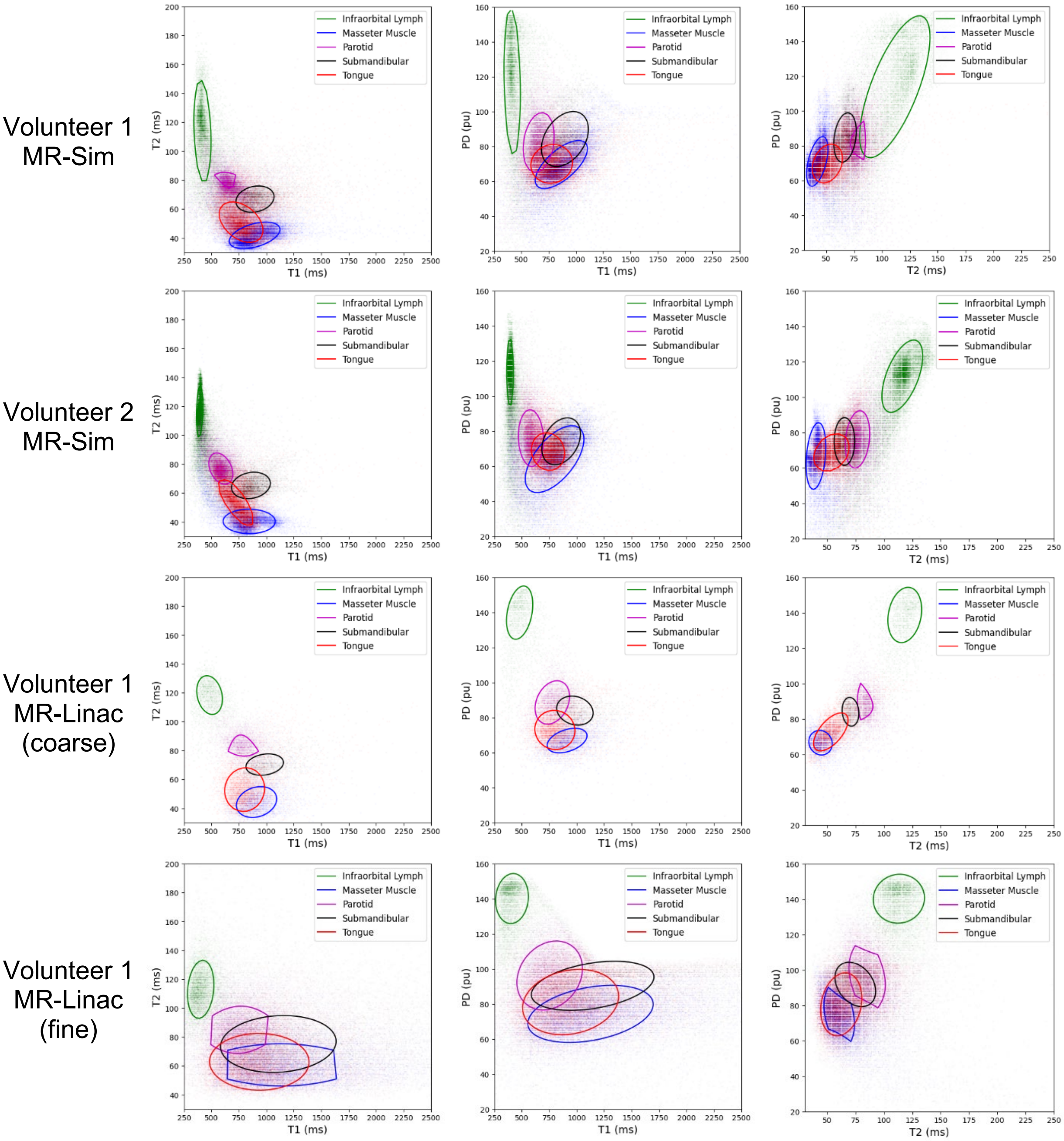

Figure 4-S1. Cluster analysis of normal tissue values for each combination of quantitative parameters (i.e., T1, T2, and PD) on both the MR-Sim and MR-Linac.
